# Hesperidin suppressed metastasis, angiogenesis and tumour growth in Balb/c mice model of breast cancer

**DOI:** 10.1111/jcmm.17902

**Published:** 2023-08-15

**Authors:** Elham Shakiba, Ali Bazi, Hamed Ghasemi, Reza Eshaghi‐Gorji, Seyyed Alireza Mehdipour, Banafsheh Nikfar, Mohsen Rashidi, Sepideh Mirzaei

**Affiliations:** ^1^ Department of Biochemistry, Faculty of Biological Sciences, North Tehran Branch Islamic Azad University Tehran Iran; ^2^ Department of Hematology Kerman University of Medical Sciences Kerman Iran; ^3^ Faculty of Allied Medical Sciences Zabol University of Medical Sciences Zabol Iran; ^4^ Student Research Committee Mazandaran University of Medical Sciences Sari Iran; ^5^ Pars Advanced and Minimally Invasive Medical Manners Research Center, Pars Hospital Iran University of Medical Sciences Tehran Iran; ^6^ Department of Pharmacology Mazandaran University of Medical Sciences Sari Iran; ^7^ The Health of Plant and Livestock Products Research Center Mazandaran University of Medical Sciences Sari Iran; ^8^ Department of Biology, Faculty of Science, Science and Research Branch Islamic Azad University Tehran Iran

**Keywords:** angiogenesis, breast cancer, hesperidin, metastasis, pathology

## Abstract

Considering the unfavourable response of breast cancer (BC) to treatment, we assessed the therapeutic potential hesperidin in mice bearing 4T1 BC tumours. Anti‐tumour effects were assessed by measuring pathologic complete response (pCR), survival analysis, immunohistochemistry for E‐cadherin, VEGF, MMP9, MMP2 and Ki‐67, serum measurement of IFNγ and IL‐4, and gene expression analysis of CD105, VEGFa, VEGFR2 and COX2. Survival of tumour‐bearing mice was the highest in mice receiving a combination of hesperidin and doxorubicin (Dox) (80%) compared to the normal saline (43%), hesperidin 5 (54%), 10 (55.5%), 10 (60.5%) and 40 (66%) mg/kg, and 10 mg/kg Dox‐treated (73%) groups (*p* < 0.0001 for all). Compared to the normal saline group, there was a significant elevation in IFNγ level in the animals receiving 20 (*p* = 0.0026) and 40 (*p* < 0.001) mg/kg hesperidin, 10 mg/kg Dox (*p* < 0.001), and combined hesperidin (20 mg/kg) and Dox (10 mg/kg) (*p* < 0.001). A significant reduction in the gene expression of CD 105 (*p* = 0.0106), VEGFa (*p* < 0.0001), VEGFR2 (*p* < 0.0001), and Cox2 (*p* = 0.034) and a significant higher pCR score (*p* = 0.006) were noticed in mice treated with 10 mg/kg Dox + 20 mg/kg hesperidin compared to those treated with 10 mg/kg Dox alone. Immunohistochemical staining showed significant reductions in Ki‐67 (*p* < 0.001) and VEGF (*p* < 0.001) and a significant elevation in E‐cadherin (*p* = 0.005) in the 10 mg/kg Dox + 20 mg/kg treatment group than in 10 mg/kg Dox alone group. Hesperidin can be considered as a potentially suitable anti‐cancer agent for BC that can synergize with other chemotherapeutics.

## 1 INTRODUCTION

Among other malignancies, women are more likely to develop tumours in their breasts, and breast cancer (BC) accounts for 33% of all women's cancers and is responsible for about 20% of cancer‐related deaths (the second leading cause) among females.^1^, ^2^, ^3^ This group of tumours show great heterogeneity in terms of response to therapy and biological specifications. In terms of histopathology, BC tumours are primarily categorized based on the expression of receptors for progesterone (PR), oestrogen (ER) and ERBB2 (also known as Her2). The classification of BC has not stopped on histopathology, extending toward molecular features and genomics and transcriptomics signatures, according to which at least 10 subtypes of BC have been characterized.^4^ Around 15% of all BC cases show negative expression for all PR, ER and Her2 (i.e. triple negative) and are known to have an aggressive behaviour, a poor response to conventional treatments, and an adverse immunologic profile with a high rate of lymphocytic infiltration, as an indicator of metastasis.^5^ Similar to many cancers, multiple triggering causative agents are considered to play a role in BC development from advanced age and intrinsic predisposing parameters to environmental triggers. Drug resistance is a rising issue in BC and is a main cause of patient death.^6^, ^7^ Surgery and radiotherapy that are used to eradicate localized tumours are ineffective in preventing metastases.^8^, ^9^ Although chemotherapy is one of the most usual therapeutic methods, due to the lack of selective cytotoxicity, this treatment is associated with many side effects. On the other hand, many cancers are resistant to chemotherapy; therefore, it is essential to find new treatment strategies with fewer side effects.^1^, ^10^

Herbal compounds with antioxidant activity have acquired great attention in the field of cancer therapy.^11^ The role of natural compounds available in the diet, especially flavonoids, in inhibiting carcinogenesis and cancer treatment has been highlighted.^11^ Flavonoids are bioactive compounds that include about 60% of the polyphenolic compounds found in plants and are abundantly found in fruits, vegetables, seeds, nuts and beverages such as tea.^12^ In addition to antioxidant, anti‐inflammatory, anti‐hypertensive and anti‐allergic effects, flavonoids have anti‐cancer properties through interference in the three stages of carcinogenesis.^13^, ^14^

Hesperetin‐7‐O‐rutinoside, or shortly hesperidin, as the most abundant flavonoid in citrus fruits, is a non‐toxic and non‐allergic flavanone glycoside with no adverse side effects.^13^, ^14^ In recent years, hesperidin's biological and pharmacological effects have been studied.^15^, ^16^ Studies show the influential role of hesperidin in improving cardiovascular function and reducing blood lipids and inflammatory markers. Also, various studies have confirmed hesperidin's antibacterial, antifungal, and antiviral role. In addition, hesperidin has anti‐cancer properties.^13^, ^14^, ^15^, ^16^, ^17^ Experiments have suggested anti‐cancer effects for hesperidin, which are supposed to be mediated through inhibiting carcinogenesis, as observed in skin and bladder cancers in animal models, as well as through suppressing tumour growth and proliferation and promoting programmed cellular death (e.g. in colon, breast and prostate cancer cells).^16^, ^18^, ^19^ Here, we created subcutaneous breast tumours in Balb/c mice and then injected different doses of hesperidin to assess its tumour growth inhibitory effects.

## 2 MATERIAL AND METHODS

### 2.1 Cell culture and 4T1 tumour bearing mice

Highly tumorigenic 4T1 mammary carcinoma cells (Pulaski &Ostrand‐ Rosenberg, 2001) were purchased from the Pasteur Institute of Iran (IPI), the National Cell Bank of Iran (NCBI), and cultured in RPMI1640 (Gibco) containing 10% fetal bovine serum (FBS) (Gibco), 100 U/mL penicillin and 100 ng/mL streptomycin (Gibco) along with mouse stem cells and lymphocytes. Under 37°C, 95% humidity and 5% CO_2_, these cells grew until reaching adequate fluency and then were detached from the floor by adding trypsin. Around 8 × 10^5^ 4T1 cells per 100 μL of RPMI1640 4T1 were subcutaneously injected to mice to develop tumours. Animals (6–8‐old‐week) who had clear tumours after 2 weeks (*n* = 42) were divided into seven experimental groups (*n* = 6 per group) as follows.

### 2.2 Experimental groups and treatments

**FIGURE 1:**
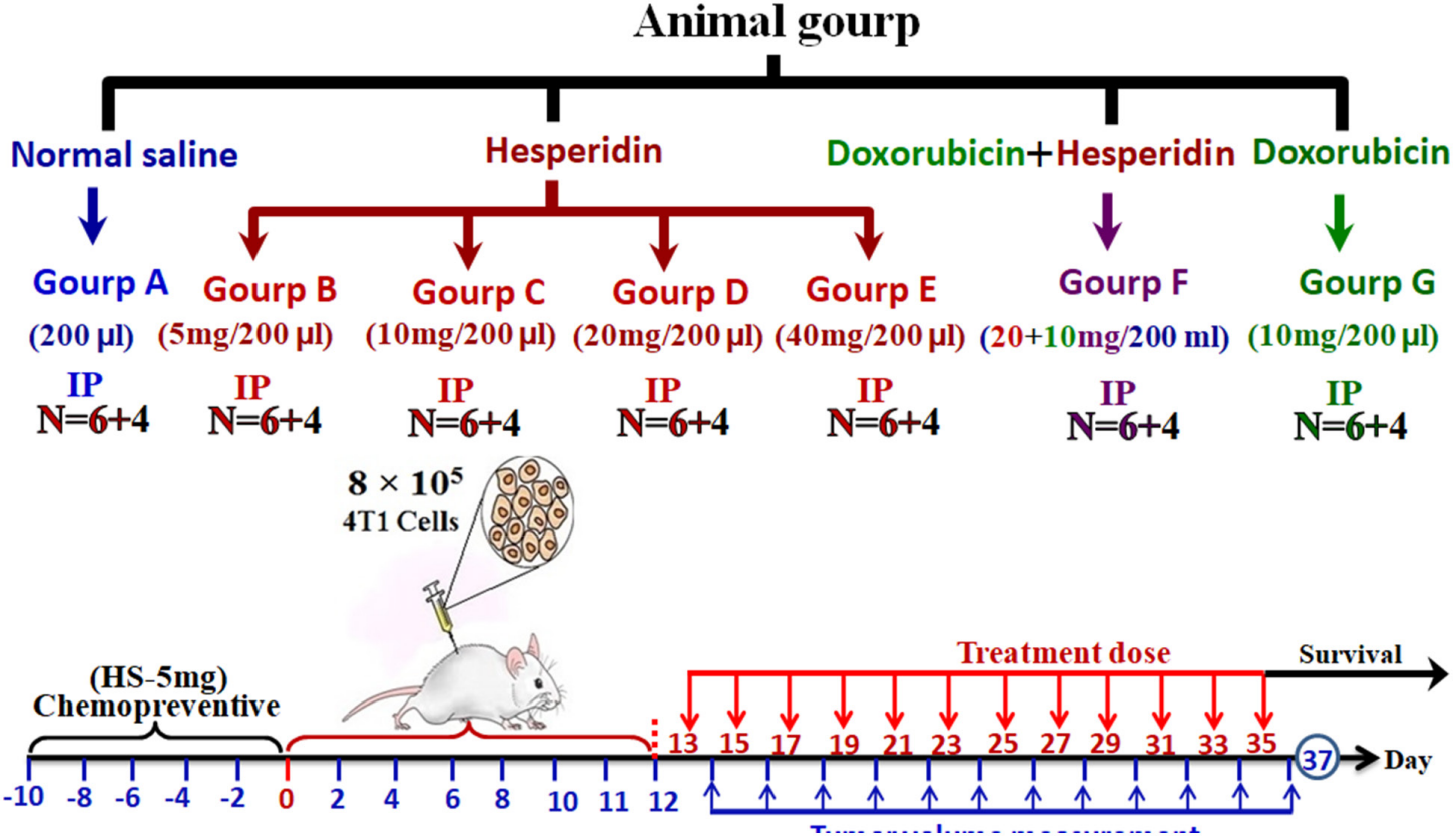
Classification of mice in the study groups.

The seven study groups included normal saline (200 mL), hesperidin (at doses 5, 10, 20, and 40 mg/200 mL), doxorubicin (Dox) (20 mg/200 mL) and hesperidin (20 mg/200 mL) + Dox (20 mg/200 mL) (Figure 1).

**FIGURE 2:**
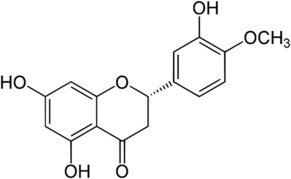
Chemical structure of hesperidin.

Twelve days after tumour induction, mice started to daily receive hesperidin (Figure 2), Dox, or normal saline for 24 days (i.e., from day 13 to day 35). Then, 24 h after the final dose (i.e. day 37), the animals were euthanized. The doses for hesperidin and doxorubicin in individual and combination therapy were selected according to previous studies.^20^, ^21^, ^22^

### 2.3 Cell proliferation assay

The 4T1 cells plus mouse lymphocytes and mouse stem cells were cultured in RPMI in a 96‐cell plate (1.2 × 10^2^ cells in each well). In order to evaluate the cytotoxicity of hesperidin, the concentrations of 100, 50, 25, 12.5, 6.25 and 3 μg/mL were obtained by the serial dilution method. Hesperidin (Sigma‐Aldrich) with a concentration of 1 M was prepared in dimethyl sulfoxide (DMSO, Merck, Germany), which as a stock solution was used to prepare target concentrations for 24‐, 48‐ and 72‐h treatments for the MTT assay. The highest amount of DMSO was always kept below 0.25%, including in the control group. After 24, 48 and 72 h of treatment, the MTT reagent [3‐(4, 5‐dimethylthiazol‐2‐yl)‐2,5‐diphenyltetrazoliumbromide], which is converted into a formazan product by living cells, was added for 4 h, and then absorbance at 570 nm was read.

### 2.4 Measurement of serum cytokines

Centrifugation (3000 rpm) for 20 min was used to separate sera, which were frozen (−70°C) until use. Serum IFNγ and IL4 concentrations were measured by commercial ELISA kits (Bioas‐say Technology). The substrate, o‐phenylenediamine, which is converted by horse radish peroxidase to a colour substance, was added, and absorbance at 492 nm was recorded using an Anthos 2020 microplate reader (Anthos, Wals, Austria). All measurements in serum were performed in duplicates, and intra‐ and inter‐assay CVs for IFNγ and IL4 were <10% and <12%, respectively.

### 2.5 Tumour size and body weight

On day 14, the mice were started to be weighed every 2 days until day 36. Tumour dimensions (L: length and W: width) were determined at 2‐day intervals in parallel with weight measurements. Tumour volume calculation was as follows:

Tumour volume=1/2×L×W2

### 2.6 Haematoxylin and eosin staining

Resected tumour sections were immersed in formalin and embedded in paraffin. Tissue blocks with 5‐μm thickness were prepared, stained with haematoxylin and eosin (H&E), and visualized under a light microscope. The pathologic complete response (pCR) scoring system, which is based on the rates of pleomorphism, mitosis, necrosis and residual tumour cells, was employed to determine response to treatment. The categories of pCR were as no response (*R* = 0, reflecting no evident reduction in the quantity of tumour cells), partial‐weak response (*R* = 1, fibrosis of tumour cells = 30%), partial‐moderate response (*R* = 2, fibrosis of tumour cells = 70%) and complete response (*R* = 3, no residual tumour cells).

### 2.7 Gene expression measurement

Tumour content of RNA was extracted using Trizol (GeneAll, Korea), which was subjected to quality and quantity assessments using agarose gel visualization and measuring the A260/A280 ratio (NanoDrop 1000 Spectrophotometer, Wilmington, DE, USA). Complementary DNA was generated using Two‐Strand Synthesis kit (General, Korea), and gene expression analysis was performed using real‐time PCR. Each PCR cycle included a 10‐s denaturation phase at 95°C, followed by a 10‐s annealing step at 55–62°C and a 30‐s extension phase at 72°C. Each PCR reaction contained template cDNA (1 μL), specific pairs of primers (1 μL), deionized water (10.5 μL) and SYBR® Green Master Mix‐Plus (Yektatajhiz, Iran) (12.5 μL) performed in Corbett RG6000 thermocycler (Australia). GAPDH was employed as the internal control gene, and the Rotor‐Gene 6000 Series Software was used to calculate the cycling threshold (CT) (Table 1). The CT (Cq) obtained was adjusted according to the respective value obtained for the internal control gene using the following formula: Cq^target^ − Cq^GAPDH^ = ΔCq. Finally, 2^(−ΔΔCq)^ formula was used to calculate gene expression in the sample (Livak & Schmittgen, 2001), where ΔΔCq = ΔCq^target^ – ΔCq^control^.

**TABLE 1:** The sequences of the forward and reverse primers used to amplify target genes by real‐time PCR.

Primer name	Primer sequence (5′ → 3′)
VEGFa	F: TCGCTCCTCCACTTCTGAGG R: GGCCATTACCAGGCCTCTTC
VEGFR2	F:AGATGCATTGTGCTGGCTCT R: AACTCGCCTGTAACCCGACT
COX	F:TCCGGAGGGAAGAGTGGAGGAAAC R:GCTGTCATTCCAGGGTCGCACAT
CD‐110	F: TCCACCAGGTGAGAAGAGTGATG R: TCACGCTCTCCAGAGTCCCATG
GAPDH	F: AACGACCCCTTCATTGAC R: TCCACGACATACTCAGCAC

### 2.8 Immunohistochemistry

The expressions of E‐cadherin (PM 170 AA Biocare), MMP9 (Abcam, ab38898), MMP2 (Abcam, ab37150), VEGF (Abcam, ab46154) and Ki‐67 (Abcam, ab15580) were evaluated using specific primary antibodies according to the manufacturer's instructions. The results were then interpreted by an experienced pathologist. For this purpose, Ki‐67 immunohistochemical staining in tumours was assessed by quantifying positive active nuclei. The expression of VEGF, MMP2, MMP9 and E‐Cadherin was determined according to the Allred scoring system, where the score of 0 indicated zero number of stained cells; score of 1 reflected <1/100 stained cells; score of 2 reflected the number of stained cells 1/100≤ and <1/10; score of three indicated stained cells 1/10≤ and <1/3; score of four indicated stained cells = 1/3 and <2/3, and score of five indicated stained cells >2/3. The intensity of staining was categorized as negative, weak, intermediate, and strong with the respective scores of 0, 1, 2 and 3. The Allred scores were considered 0–1, 2–3, 4–6 and 7–9 meaning non‐reactive, weak, intermediate and high reactivity, respectively.

### 2.9 Statistical analysis

Means ± standard errors (SEM) with 95% confidence intervals were used to describe the results using GraphPad Prism 5 software (La Jolla, CA) for Windows. One‐way analysis of variance, followed by the Tukey's post‐hoc test, at a significant level of *p* value ≤0.05, was used to compare groups.

### 2.10 Ethical considerations

Standard ethical protocols in working with laboratory animals were followed to ensure minimal harm and inconvenience to animals. Euthanizing was performed after taking the mice away from other animals. In addition, the methods were reviewed and approved by the university's ethical committee for working with laboratory animals.

## 3 RESULTS

### 3.1 Body weight and tumour volume changes

**FIGURE 3:**
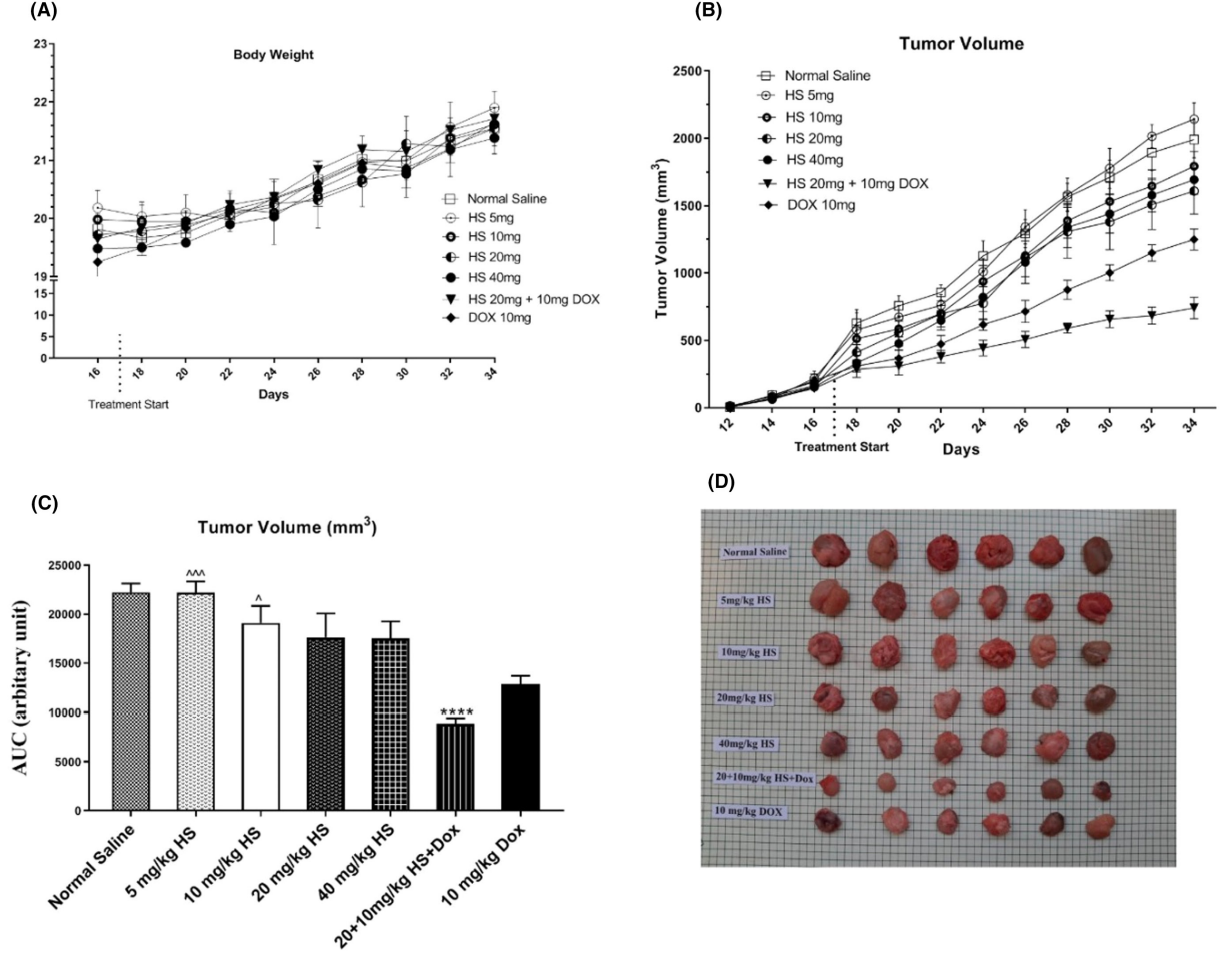
Body weight (A) and tumour volume (B–D) alterations in tumour‐bearing mice treated with hesperidin, normal saline or doxorubicin. The values represent mean ± standard error (SEM) from six measurements. The signs * and ^ represent *p* < 0.05 compared to normal saline and doxorubicin, respectively.

Body weight was measured in 2‐day intervals during the study. Average body weight did not significantly change in the study groups during the study and body weight changes were comparable in animals in different groups during the study (Figure 3B–D), showing no significant difference between the group receiving hesperidin, normal saline or Dox.

However, the mean tumour volume in hesperidin‐treated mice (at doses of 10, 20 and 40 mg/kg) on days 28, 30 and 34 was significantly (*p* < 0.01) lower than that in the normal saline group. In addition, the hesperidin + Dox group on days 20 and later had a significantly lower tumour volume than the normal saline group (*p* < 0.01). The graph of the area under the curve (Figure 3C) showed that the hesperidin + Dox group had a lower tumour surface than the normal saline group during the study. The macroscopic images of tumours extracted from mice have been shown in Figure 3D.

### 3.2 Cell viability

**FIGURE 4:**
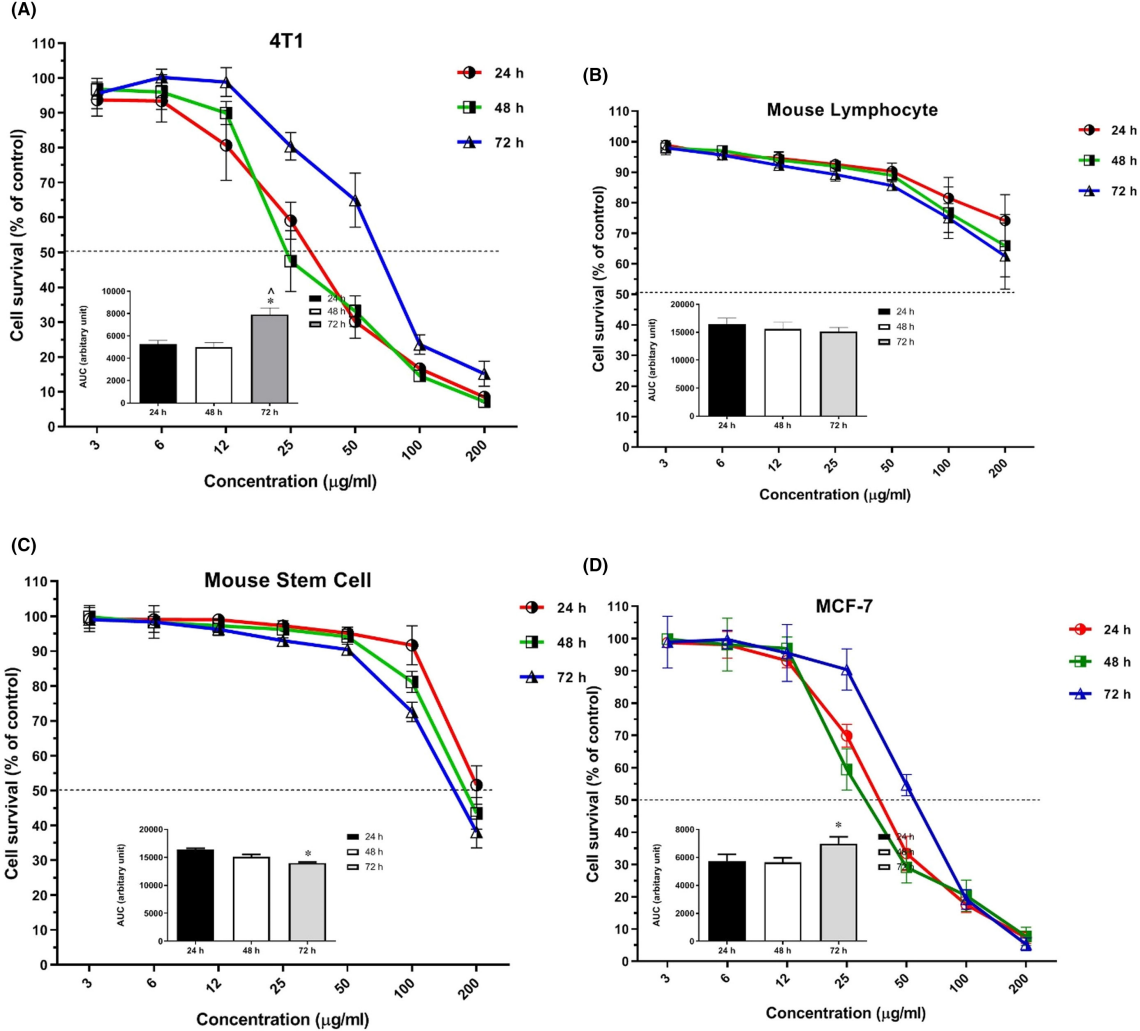
After 24, 48 and 72 h of treatment with hesperidin, IC50 values were calculated for 4T1 cells (A), mouse‐derived lymphocytes (B), mouse bone marrow‐derived stem cells (C), and MCF‐7 cells (D). The signs * and ^ represent *p* < 0.05 in comparison with normal saline and doxorubicin, respectively.

After 72‐h of treatment with hesperidin, the survival of tumour cells fell below 50%, showing a significant difference compared to 24‐ and 48‐h treatment groups (*p* = 0.008). Lymphocyte count, as an indicator of metastasis, was higher than in the 24‐h treatment group than in other two groups. Figure 4 and Table 2 show the IC50 values obtained for hesperidin at different times. Another noteworthy point is that none of the groups reached survival below 50% up to a concentration of 200 μg/mL of hesperidin. Moreover, the number of stem cells in the 24‐h group was more than in the other two groups. Also, the decrease in the number of stem cells in the 72‐h treatment group was statistically significant compared to other groups (*p* = 0.0032).

**TABLE 2:** IC50 values of hesperidin against mouse derived peripheral blood monuclear cells, mouse bone marrow stem cells, MCF7 human breast cancer cell line and 4T1 mouse breast cancer cells.

Cell line	Mean IC50 ± SD (95% CI) (μg/mL)	Mean IC50 ± SD (95% CI) (μg/mL)	Mean IC50 ± SD (95% CI) (μg/mL)
	24 h	48 h	72 h
4T1	25.4 ± 2.9 (21–29.4)	33.3 ± 3.6 (30.6–38.8)	66.37 ± 2.5 (61.6–61.6)
MCF7	27.9 ± 3.1 (24.5 to 32.6)	31.6 ± 2.6 (27 to 34.2)	63.2 ± 3.8 (59.6 to 67.2)
Mouse BMDSCS	302.4 ± 4.5 (199.8–313.4)	196.2 ± 7 (169–194.5)	159 ± 7.3 (145.4–173.9)
Mouse PBMCs	4651 ± 3670.7 (2603–16,112)	2203 ± 1883.3 (1244–8211)	901.1 ± 143.2 (498.6–1048)

### 3.3 Survival of tumour‐bearing mice

**FIGURE 5:**
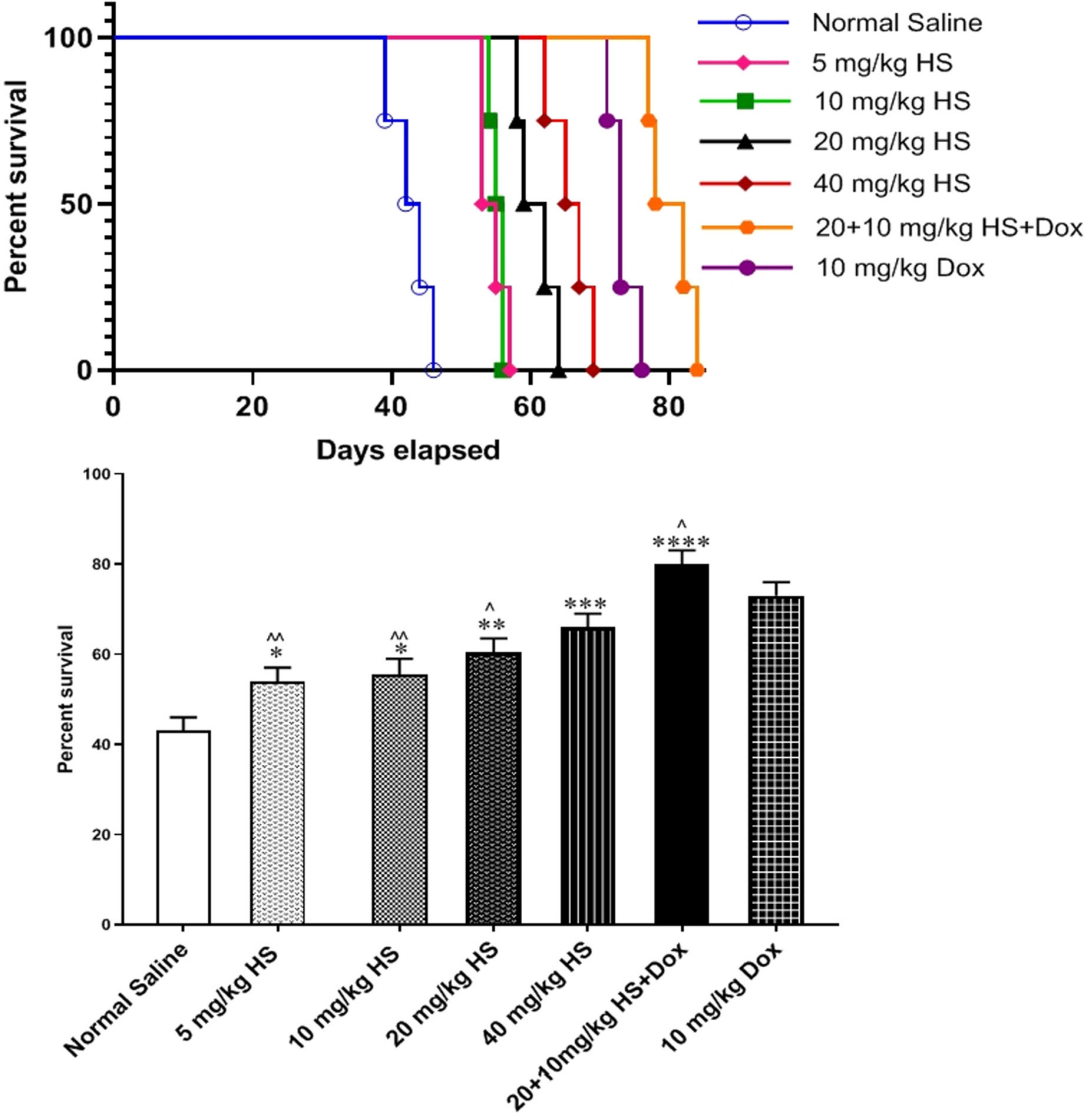
Survival of tumour‐bearing mice after treatment with different doses of hesperidin, either alone or along with doxorubicin (Dox). *n* = 3 per group. The signs * and ^ represent *p* < 0.05 compared to normal saline and doxorubicin, respectively.

Survival in normal saline treated mice fell below 50% (43%), which was significantly lower compared to the animals treated with hesperidin 5 (54%), 10 (55.5%), 10 (60.5%) and 40 (66%) mg/kg. However, survival percentage was significantly higher in 10 mg‐Dox‐treated animals (73%) compared to hesperidin‐treated mice (*p* < 0.001 for all). Finally, mice receiving a combination of hesperidin and Dox showed a significantly higher survival percentage (80%) compared to the normal saline (43%) and Dox‐treated (73%) (*p* < 0.001 for both comparisons). Median survival in drug‐treated and normal saline‐treated animals have been shown in Figure 5.

### 3.4 Hesperidin effects on inflammatory cytokines

**FIGURE 6:**
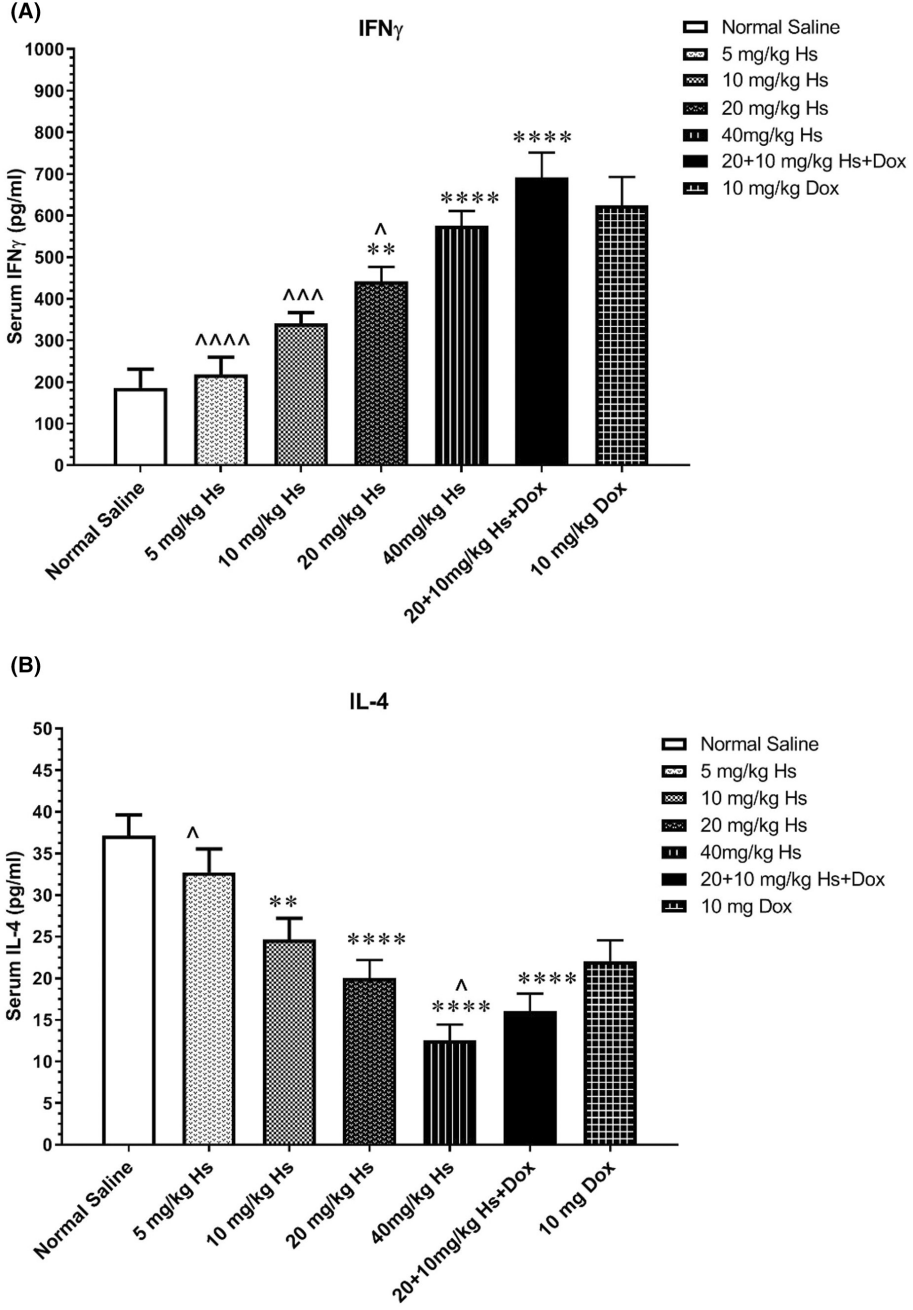
Effects of hesperidin on serum levels of (A) IFNγ and (B) IL‐4 in Balb/c mice with breast tumours. The values are mean ± standard error (SEM) of six measurements. * and ^: *p* < 0.05; ** and ^^: *p* < 0.01; and *** and ^^^: *p* < 0.001. The signs * and ^ represent in comparison with the normal saline and doxorubicin groups, respectively.

As shown in Figure 6, hesperidin increased the level of IFNγ in a dose‐dependent manner. Compared to the normal saline group, there were significant elevations in IFNγ level in the animals receiving 20 (*p* = 0.0026) and 40 (*p* < 0.001) mg/kg hesperidin, 10 mg/kg Dox (*p* < 0.001), and combined hesperidin (20 mg/kg) + Dox (10 mg/kg) (*p* < 0.001).

Serum IL‐4 level showed a significant reduction in the animals treated with 10 mg/kg Dox compared to normal saline group (*p* < 0.0001) and 5 mg/kg hesperidin but not compared to 10 and 20 mg/kg groups. Also, IL‐4 serum level was significantly lower in the groups of 40 mg/kg hesperidin and combined Dox and hesperidin compared to animals treated with 10 mg/kg Dox alone.

### 3.5 Effect of hesperidin on mRNA expression of target genes

**FIGURE 7:**
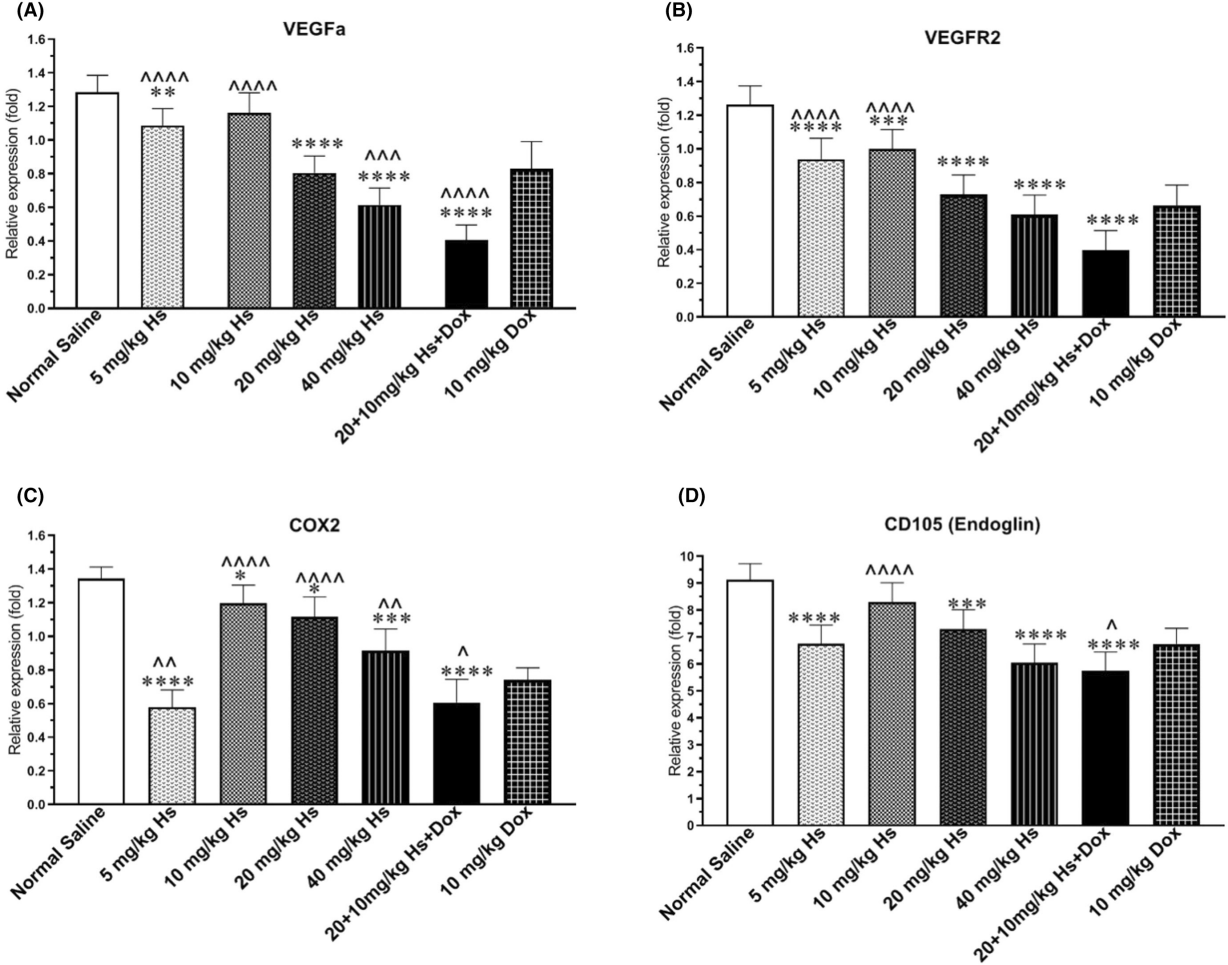
Real‐time PCR experiment to determine the expression of a number of genes involved in angiogenesis and inflammation in Balb/c mice carrying 4T1 breast tumours after receiving different doses of hesperidin. Values represent mean ± standard error (SEM) of six measurements. * and ^: *p* < 0.05; ** and ^^: *p* < 0.01; ^^^: *p* < 0.001. The signs * and ^ represent in comparison with the normal saline and doxorubicin groups, respectively.

Figure 7 shows the expression levels for the genes analysed.

#### 3.5.1 VEGFa

The expression of VEGFa gene significantly decreased in Dox‐treated animals compared to normal saline group, and hesperidin‐treated mice at the doses of 5 and 10 mg/kg (*p* < 0.0001 for all comparisons) but not compared to animals receiving 20 mg/kg hesperidin. On the other hand, the expression of this gene was significantly lower in the 40 mg/kg hesperidin (*p* = 0.0004) and combined hesperidin + Dox (*p* < 0.0001) compared to Dox‐treated group (Figure 7A).

#### 3.5.2 VEGFR2

The downregulation of the VEGFR2 gene in the 10 mg/kg Dox‐treated group was statistically significant compared to the normal saline, and 5 and 10 mg/kg hesperidin groups (*p* < 0.0001 for all comparisons) but it was comparable with the 20 and 40 mg/kg hesperidin groups. Also, the expression of this gene was significantly lower in the combined treatment group compared to the group receiving Dox alone (*p* < 0.0001, Figure 7B).

#### 3.5.3 COX2

The mice treated with 10 mg/kg Dox showed significantly lower expression of the COX2 gene compared to the normal saline (*p* < 0.0001) and hesperidin‐treated groups at the doses of 5, 10, 20, and 40 mg/kg (*p* values of 0.0069, <0.0001, <0.0001 and 0.0032, respectively). Also, the expression of this gene was significantly lower in the combined treatment group compared to the Dox‐treated group (*p* = 0.034, Figure 7C).

#### 3.5.4 CD105

Regarding the expression of CD 105, a significant reduction was observed in mice treated with 10 mg/kg Dox (*p* < 0.0001) compared to normal saline group and 10 mg/kg hesperidin group (*p* < 0.0001); however, this reduction was comparable between Dox‐treated and hesperidin‐treated (5, 20, and 40 mg/kg) animals. The downregulation of CD105 was statistically significant in the combination therapy group (i.e. Dox + hesperidin) compared to Dox‐treated animals (*p* < 0.0106, Figure 7D).

### 3.6 Pathologic analysis of 4T1 mammary tumours

**FIGURE 8:**
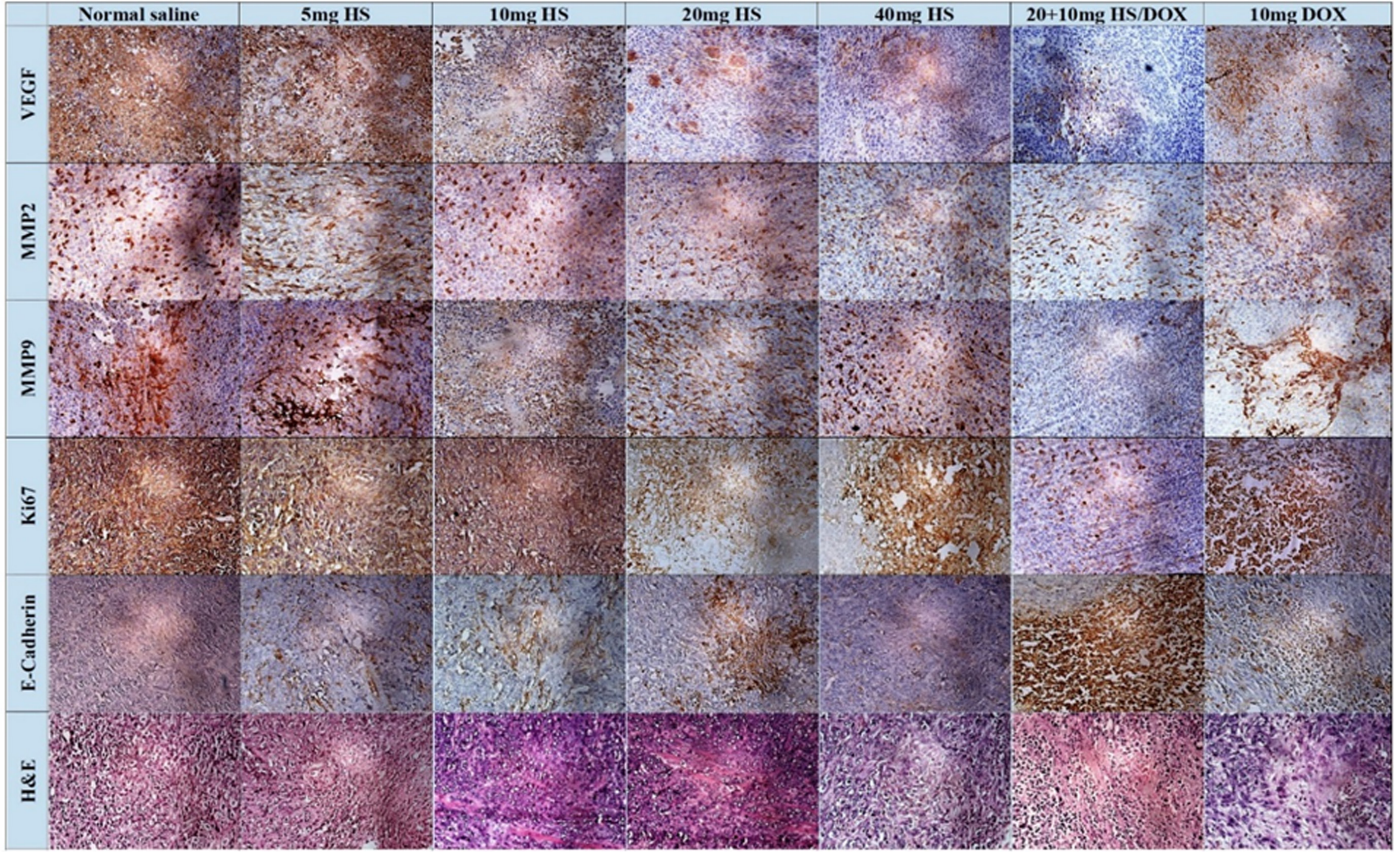
Breast tumour tissues (4T1) were harvested from mice and stained by haematoxylin and eosin (H&E) for histological examinations. Primary antibodies for MMP9, MMP2, E‐cadherin, Ki‐67 and VEGF were used to determine the expression of these markers in tumours after treatment with hesperidin, doxorubicin or normal saline.

**FIGURE 9:**
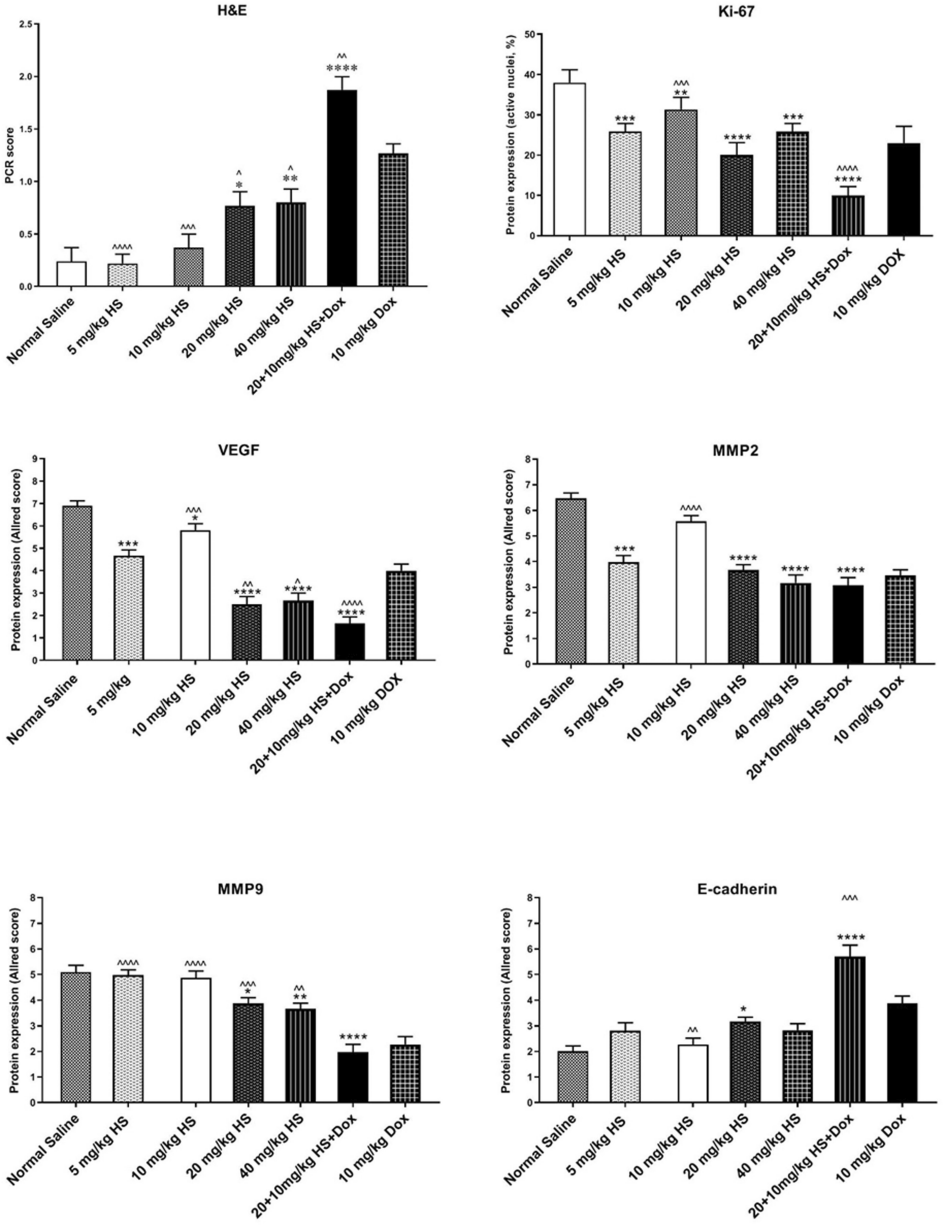
Assessment of pathologic complete response (pCR) in the experimental groups. The expression of Ki‐67 was evaluated based on the percentage of active nuclei and positive cells. The intensity of E‐Cadherin, MMP9, MMP2 and VEGF was determined using the Allred scoring system. Values represent mean ± standard error (SEM) of six measurements. * and ^, *p* < 0.05; ** and ^^, *p* < 0.0;, ^^^, *p* < 0.001. The signs * and ^ represent in comparison with the normal saline and doxorubicin groups, respectively.

As shown in Figures 8 and 9A, compared to the normal saline‐treated group, hesperidin groups (at doses 20 (*p* = 0.02) and 40 mg/kg (*p* = 0.012)) showed significantly higher pCR scores. Also, pCR was significantly higher in Dox (10 mg) compared to 10 (*p* < 0.001), 20 (*p* < 0.01), and 40 (*p* = 0.032) mg/kg of hesperidin. Moreover, the combinational treatment of hesperidin + Dox delivered a significantly higher pCR score than the Dox group alone (*p* = 0.006) and normal saline (*p* < 0.001) (Figures 8 and 9A).

### 3.7 Findings of IHC

The results of immunohistochemical staining have been shown in Figure 8.

#### 3.7.1 Ki‐67

Ten fields were randomly observed at 400x and 100 nuclei were counted in each field to obtain the ratio of immunoreactive nuclei. The expression of Ki‐67 was significantly lower in hesperidin‐treated groups at the doses of 5 (*p* < 0.001), 10 (*p* = 0.002), 20 (*p* < 0.001) and 40 mg/kg (*p* < 0.001) compared to the normal saline group. Furthermore, Ki‐67 expression was significantly lower in the hesperidin + Dox group compared to Dox or normal saline alone (*p* < 0.001 for both).

#### 3.7.2 VEGF

The expression of VEGF was significantly lower in hesperidin‐treated groups at the doses of 5, 10, 20, and 40 mg/kg and combined hesperidin + Dox compared to the normal saline group (*p* < 0.001 for all). Compared to the group treated with 10 mg Dox, VEGF expression was significantly lower in the groups treated with 20 (*p* = 0.005) and 40 (*p* = 0.016) mg/kg hesperidin alone and combined hesperidin + Dox (*p* < 0.001).

#### 3.7.3 MMP2

Compared to the normal saline group, the groups treated with hesperidin (5 mg, *p* = 0.007; 20 mg, *p* < 0.001; and 40 mg, *p* < 0.001) and combined hesperidin + Dox (*p* < 0.001) showed significantly lower expression of MMP2. The expression of MMP2 was comparable between the animals receiving 10 mg/kg Dox alone and those treated with combined hesperidin + Dox.

#### 3.7.4 MMP9

The groups treated with 20 (*p* = 0.01) and 40 (*p* = 0.002) mg/kg hesperidin showed significantly lower expression of MMP9 compared to the normal saline group, but not compared to 10 mg Dox‐treated animals. The animals treated with 10 mg Dox showed significantly lower expression of MMP9 compared to those receiving hesperidin alone (5 mg, *p* < 0.001; 10 mg, *p* < 0.001; 20 mg, *p* = 0.006; and 40 mg, *p* = 0.003). The group treated with the combination of hesperidin and Dox showed significantly lower expression of MMP9 compared to the saline‐treated group (*p* < 0.001) but not compared to the 10 mg‐Dox‐treated group.

#### 3.7.5 E‐cadherin

E‐cadherin expression showed a significant upregulation in the group receiving 20 mg/kg hesperidin compared to the normal saline treated group (*p* = 0.043). Also, combination treatment with hesperidin and Dox led to a significant increase in E‐cadherin expression compared to the normal saline (*p* < 0.001) and 10‐mg‐Dox‐treated (*p* = 0.005) groups.

### 3.8 Adverse effects of hesperidin on main tissues

Tissue sections were obtained from the liver (*n* = 11, five from hesperidin‐treated and six from normal saline‐treated), lungs (*n* = 8, three from hesperidin‐treated and five from normal saline‐treated) and kidneys (*n* = 9, four from hesperidin‐treated and five from normal saline‐treated) (Table 3). The results showed that hesperidin treatment led to slight necrosis, oedema, haemorrhage and hydropic degeneration compared to the animals treated with normal saline.

**TABLE 3:** Liver, lung and kidney histopathology in healthy mice treated with hesperidin or normal saline.

Tissue	Parameter	Hesperidin	Normal Saline
Liver	Normal	5 (83.3%)	6 (100%)
	Mild necrosis	0 (0%)	0 (0%)
	Edema	0 (0%)	0 (0%)
	Hydropic degeneration	1 (16.7%)	0 (0%)
Lungs	Normal	3 (50%)	5 (83.3%)
	Oedema	3 (50%)	1 (16.7%)
	Haemorrhage	2 (33.3%)	0 (0%)
	Infiltrative cells	1 (16.7%)	1 (16.7%)
Kidneys	Normal	4 (66.7%)	5 (83.3%)
	Oedema	1 (16.7%)	1 (16.7%)
	Infiltrative cells	0 (0%)	0 (0%)

## 4 DISCUSSION

Hesperidin has been reported to have various therapeutic activities, including cardioprotective,^23^ neuroprotective,^24^ hypolipidemic,^25^ anticancer,^26^ antidiabetic,^27^ antimicrobial, anti‐inflammatory and antioxidant activities^28^ that directly reflect the therapeutic potential of hesperidin in treating numerous medical conditions such as cancer.^29^, ^30^, ^31^

Our findings showed that hesperidin boosted survival rate and decreased tumour sizes in 4T1‐tumour bearing mice. In line with our results, decreased mean growth and volume of the MG‐63 tumour were reported after 2 weeks of treatment with hesperidin, and this in vivo inhibitory effect was concentration‐ and time‐dependent.^13^ In DMBA‐induced rat models of breast cancer, hesperidin (200 mg/kg) was shown to increase survival, prevent tumour development, and reduce tumour volume.^32^

We noticed that the group treated with hesperidin (20 mg/kg) + Dox (10 mg/kg) had increased concentration of IFN‐γ and decreased concentration of IL‐4i n serum. IFN‐γ is essential for the effective stimulation of cellular immunity and subsequently stimulating the antitumor immune response. A similar study by Patel et al.^32^ in mice with breast cancer found that hesperidin (200 mg/kg) increased IFN‐γ and decreased IL‐4, which decreased the growth of cancer cells.^33^

In the 4T1 mammary tumour tissues harvested from hesperidin‐treated mice, we observed a significant downregulation in the VEGFa, VEGFR2, CD110 and COX2 genes. CD105 is a strong indicator of neovascularization in human malignancies,^34^ and VEGFa roles as a stimulator for the proliferation and migration of endothelial cells and a suppressor of apoptosis. Furthermore, VEGFa increases vascular permeability^35^ and COX‐2 stimulates the activity of cancer stem cells and enhances apoptotic resistance, proliferation and angiogenesis.^36^ In a similar study in a mouse model of breast cancer, hesperidin was reported to suppress VEGF production and tumour growth and to reduce microvessel density through suppressing the expression of VEGF, VEGFR2 and NFATc3 proteins.^37^ In addition, the inhibitory effects of hesperidin on COX‐2 in kidney cancer (100 mg/kg)^38^ and lung cancer (25 mg/kg)^39^ have previously been reported. The main difference between these studies was the effective dose of hesperidin, which was much higher than in the current study, indicating that hesperidin can exert equally significant effects at relatively lower doses as well.

We here demonstrated that hesperidin at different doses led to an increase in pCR and the ratio of cancerous cells undergoing cell death but these effects were inferior compared to doxorubicin, where the most prominent effects were observed when the combination of hesperidin and doxorubicin was used compared to the normal saline and doxorubicin alone groups.

We also observed that VEGF expression decreased following the administration of different doses of hesperidin, but this effect was less prominent in doxorubicin alone treatment group. However, combined treatment with doxorubicin and hesperidin had more prominent reducing effects than the normal saline and doxorubicin alone treatment groups. In different types of cancer, including breast cancer, VEGF seems to be involved in angiogenesis.^40^ It has been reported that hemoxygenase A (HO‐1), which degrades heme can enhance angiogenesis by increasing the expression of VEGF, contributing to tumour growth and metastasis.^41^ This function is complementary to the enzymatic remodelling of type IV basement membrane collagen by MMP‐2 and MMP‐9, two zinc‐dependent endopeptidases, which thereby are involved in promoting cancer metastasis.^42^ Flavonoids such as Quercetin and Epigallocatechin gallete can reduce VEGF expression and prevent angiogenesis in proliferating tumour tissue.^43^, ^44^ It has been reported that hesperidin modulates angiogenesis in tumours via targeting basic fibroblast growth factor (bFGF), VEGF, and MMPs.^45^ In another study, hesperidin revealed anti‐proliferative and anti‐metastatic effects against C57BL6/N mice in vivo, B16‐F10 metastatic murine melanoma cells in vitro,^46^ as well as HepG2 human hepatocellular carcinoma cells.^47^ These effects may partly be mediated by inhibiting the cytosolic expression of MMP‐9 via suppressing protein pathway activator‐1 and NF‐κB signalling. In lung cancer and hepatocellular carcinoma cells, hesperidin further reduced the expression of MMP‐9 and MMP‐2.^39^ Hesperidin effectively reduced tumour growth, which was accompanied by depressed vascular density and downregulation of VEGF, NFATc3, VEGF, and VEGFR2 in xenograft BALB/c mice model in vivo and human breast cancer cells in vitro.^37^ In the study conducted by Kongtawelert et al.^42^ in 2020, hesperidin (10–50 μM) suppressed the migration of MDA‐MB‐2 breast cancer cells via significantly reducing MMP‐2 and MMP‐9 activities. Investigating the mechanisms of the inhibitory effects of hesperidin against cancer cells using in vitro and in silico techniques^48^ showed a considerable downregulation of ALDH1, MMP‐9, Bcl‐2 and p21 accompanied by an increase in p53 and cyclin D1. Bioinformatic analysis suggested that 11 genes may contribute to the anti‐tumour effects of hesperidin, including AURKB, BCL6, CASP3, CD80, ghrelin, G6PD, HMOX1, IRF‐7, MMP9, TP53 and (SP1).^48^ In 2020, Nurhayati et al.^49^ reported that simultaneous treatment with doxorubicin and hesperidin had considerable cytotoxic, G2/M phase‐inducing, and pro‐apoptotic effects, and hesperidin (95 μM) in combination with doxorubicin (0.2 μM) had synergistic effects and reduced cell migration in MCF‐7 cells compared to doxorubicin alone, accompanied by decreased the levels of MMP‐9, HER2, and Ras 1 (Rac1) proteins.^49^ In a similar study in mice bearing breast tumours, hesperidin significantly inhibited VEGF production and angiogenesis and suppressed tumour growth via modulating the expression of VEGF, VEGFR2 and NFATc3.^37^ In another study investigating the expression of VEGFR2 after treatment with hesperidin in a mouse model of glioma, it was shown that hesperidin (20 mg/kg) could significantly inhibit the HIF‐1a/VEGF/VEGFR2 pathway.^20^

Ki‐67 and E‐cadherin are cancer prognostic and diagnostic factors related to cell proliferation.^41^, ^50^ In the current study, IHC was used to quantify the expression of Ki‐67 and E‐cadherin in tumour tissues from mice treated with hesperidin, showing a remarkably reduction in Ki‐67, while E‐cadherin increased substantially in comparison with the normal saline group. The expression of Ki‐67 in the hesperidin group, however, was still significantly higher compared to the 10 mg doxorubicin group. Also, combined hesperidin and doxorubicin treatment led to a further reduction in Ki‐67 marker. The expression of E‐cadherin was significantly higher in the 20 mg hesperidin group compared to the normal saline group and significantly lower in the 10 mg hesperidin group compared to the 10 mg doxorubicin group. Likewise, the co‐administration of hesperidin and doxorubicin led to a further increase in E‐cadherin. A decrease in Ki‐67 and an increase in E‐cadherin in hesperidin‐treated mice (alone and in combination with doxorubicin) indicate that hesperidin could be considered as a suitable agent with inhibitory effects on the proliferative and migratory properties of breast cancer cells. In line, similar studies have suggested hesperidin as a potential anti‐tumour agent against breast cancer‐related cell lines. For example, hesperidin (100 μM) was reported to significantly inhibit the proliferation of green fluorescent protein/α‐tubulin‐transfected MCF‐7 cells^18^ probably via pathways independent of the cell cycle regulatory mechanisms as minimal effects were seen on the number of mitotic cells. This observation agrees with the reports of similar experiments confirming the dose‐dependent growth inhibitory effects of hesperidin on MCF‐7^22^, ^51^ and MDA‐MB‐231,^42^, ^52^ which may be attributable, at least in part, to the suppression of programmed death ligand 1 (PD‐L1; an immune checkpoint protein)^53^ and the AKT and NF‐κB signalling pathways.^42^ Patel et al.^32^ reported that the combination of doxorubicin and hesperidin could inhibit the growth of breast cancer cells in female rats via reducing Ki67 expression compared to the animals treated with DMBA.^32^

It is noteworthy that subcutaneous models have shortcomings compared to orthotopic models in terms of heterogeneity, tumour growth condition and tumour progression.^54^, ^55^ Further studies should include an orthotopic 4T1 model, and possibly an orthotopic human xenograft model in immunocompromised mice.

## 5 CONCLUSION

Hesperidin showed significant anti‐angiogenesis and anti‐proliferative effects in mice bearing 4T1 breast cancer, suggesting this compound as a potential treatment either with or without standard treatment (doxorubicin) to be used in future clinical research. The role of hesperidin as an inhibitor of angiogenesis, tumour growth and metastasis was confirmed in this study. It is suggested to consider clinical studies to investigate the effects of this agent in humans.

## AUTHOR CONTRIBUTIONS

**Elham Shakiba:** Data curation (equal); writing – original draft (equal). **Ali Bazi:** Data curation (equal); validation (equal); writing – review and editing (equal). **Hamed Ghasemi:** Conceptualization (equal); methodology (equal). **Reza Eshaghi‐Gorji:** Writing – review and editing (equal). **Seyyed Alireza Mehdipour:** Conceptualization (equal); methodology (equal). **Banafsheh Nikfar:** Formal analysis (equal). **Mohsen Rashidi:** Conceptualization (equal); funding acquisition (equal); methodology (equal); supervision (equal). **Sepideh Mirzaei:** Data curation (equal); project administration (equal).

## CONFLICT OF INTEREST STATEMENT

The authors declare that they have no conflict of interest.

## Data Availability

The data that support the findings of this study are available from the corresponding author upon reasonable request.
